# Supplementary material for: Setdb1 and Atf7IP form a hetero-trimeric complex that blocks Setdb1 nuclear export
Source: J Biol Chem. 2025 May 6;301(6):110171. doi: 10.1016/j.jbc.2025.110171 (PMC12167477; doi:10.1016/j.jbc.2025.110171)
Supplement: Supplementary Figs [file mmc1.pdf]

Figure S1

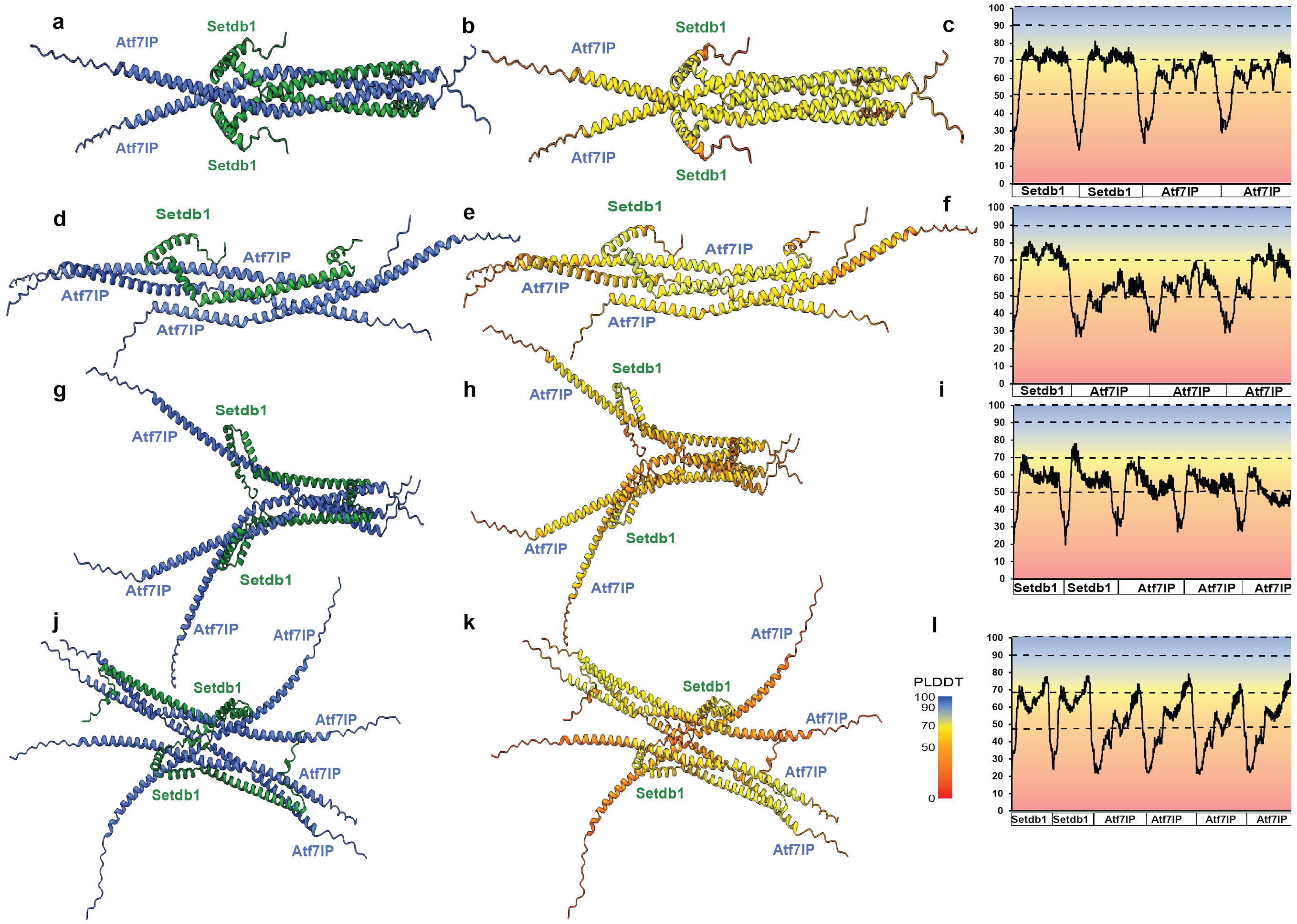

Figure S1: The low-confidence Alphafold2 models of the minimal Setdb1/Atf7IP complex with different stoichiometries. These models represent the different stoichiometries tested for the Setdb1/Atf7IP minimal interaction, other than the 1:2 interaction; a) 2Setdb1:2At7IP d) 1Setdb1: 3Atf7IP g) 2Setdb1:3Atf7IP j) 2Setdb1:4Atf7IP The right (b-c, e-f, h-i, k-l) of each panel contains the pLDDT-colored model along with its pLDDT plot predicted for each stoichiometry.

Figure S2

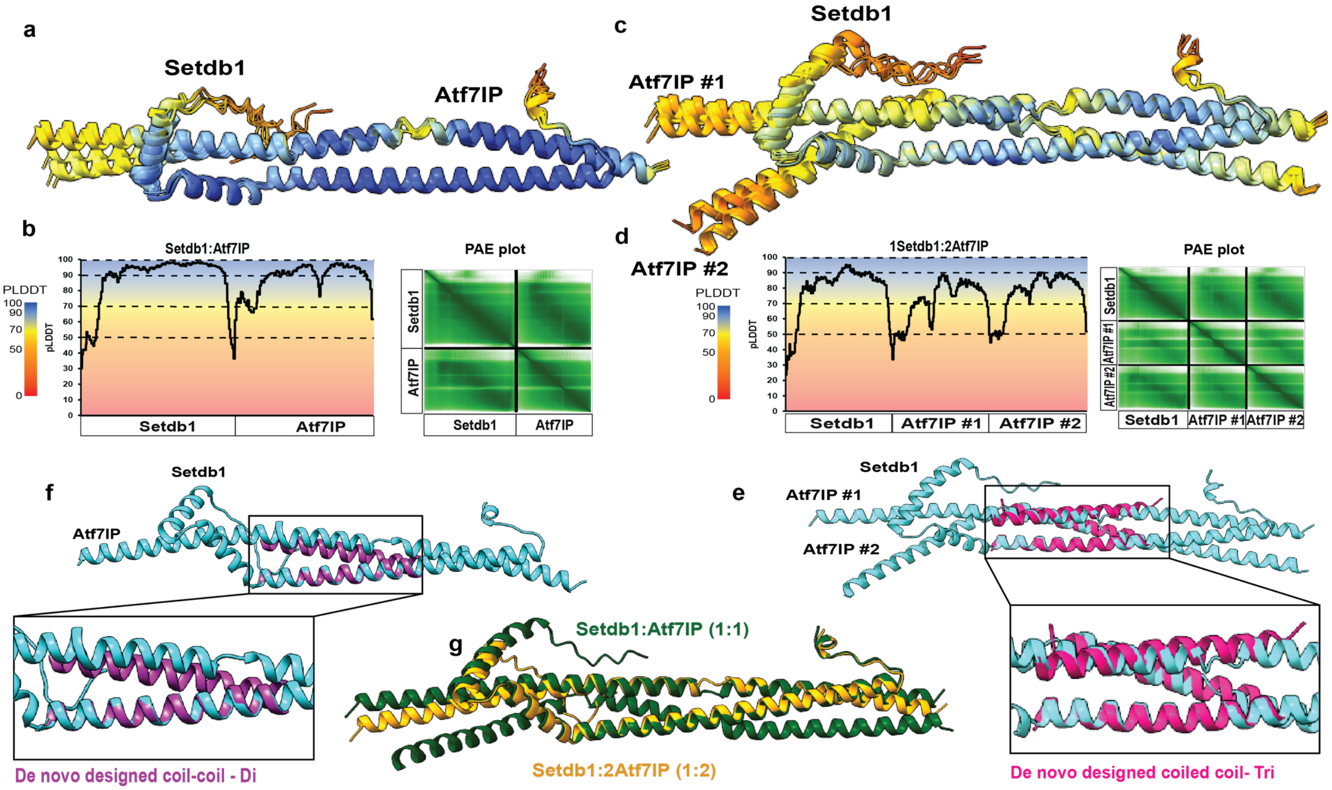

Figure S2: SETdb1/Atf7IP 1:1 AF2 model has a slightly higher pLDDT and PAE scores than its 1:2 AF2 model. a,c) Alignment of five top ranked models of the Setdb1(2-115)/Atf7IP(574-666) 1:1 and 1:2 models colored by pLDDT scoring. b) pLDDT plot and PAE plot of Setdb1/Atf7IP 1:1 highest ranked model d) pLDDT plot and PAE plot of Setdb1/Atf7IP 1:2 highest ranked model e,f) Superimposed idealized coiled-coils (pdb: 4dzl and 4dzm) with the dimeric and hetrotrimeric models of the Setdb1/Atf7IP minimal complex respectively. g) Superimposed model of the 1:1 and 1:2 Setdb1/Atf7IP minimal complexes.

Figure S3

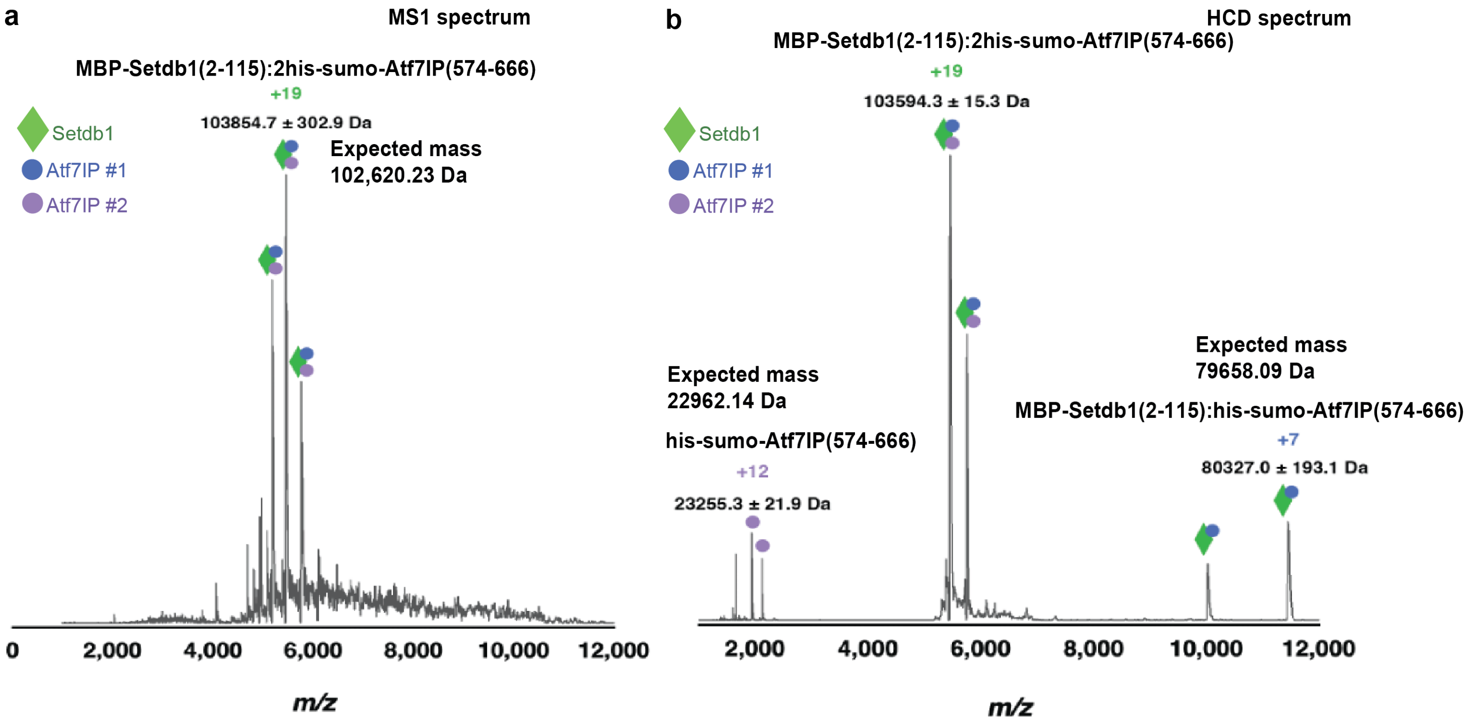

Figure S3 Native mass spectrometry validates heterotrimeric stoichiometry of the MBP-SETdb1(2-115)/HS-Atf7IP(574-666) complex.
a) Raw MS1 spectrum obtained on UHMR showing charge state distribution of possible 1 MBP-Setdb1(2-115): 2(6Xhis-sumo-Atf7IP(574-666) complex. b) HCD 220 spectrum following isolation of the 5300-5800 m/z. Most abundant charge states in each distribution are labeled. The values reported are from an average of three measurements. Representative spectrum from a single measurement shown.

Figure S4

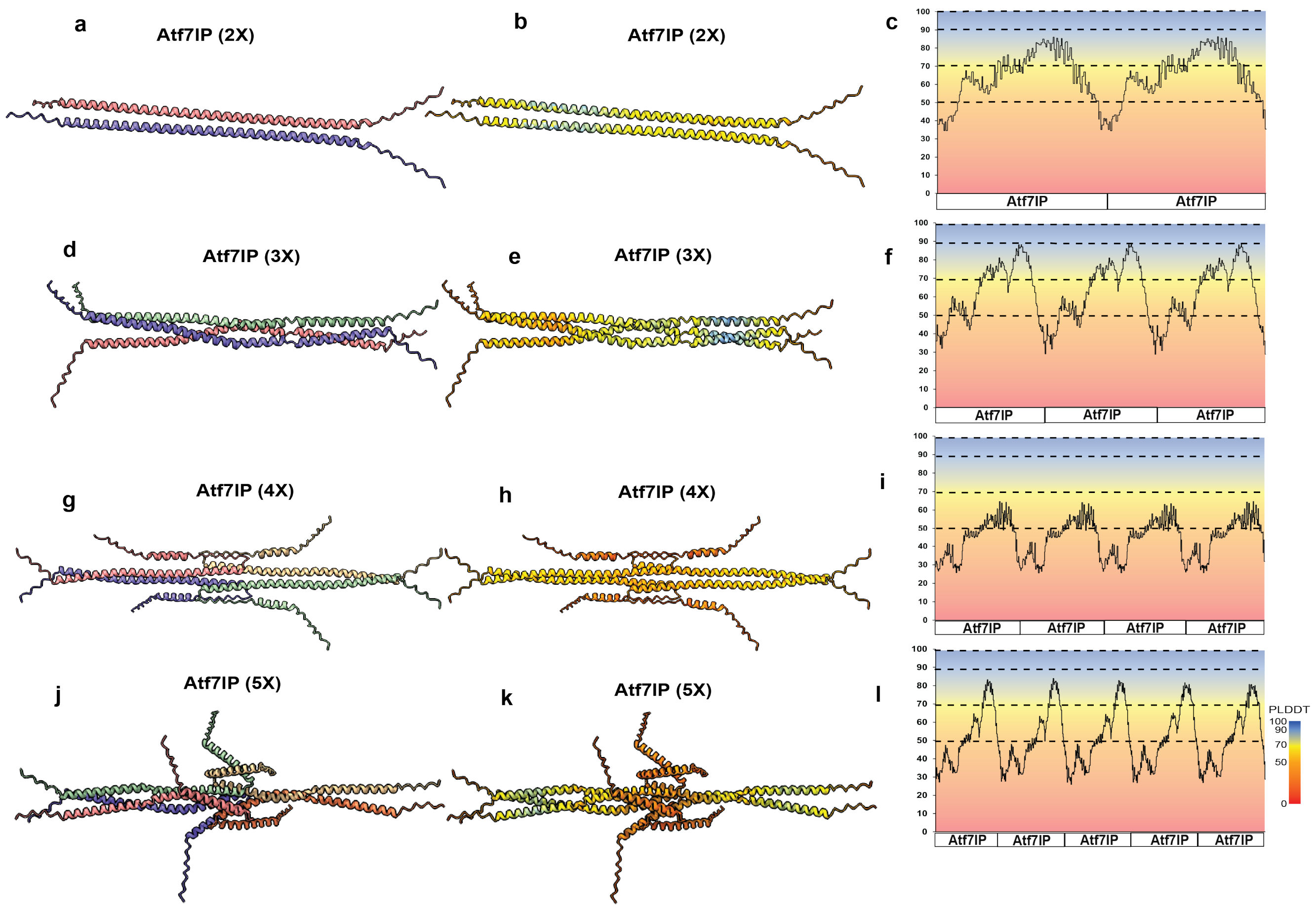

Figure S4 Alphafold2 models of Atf7IP(574-666) fragments with itself do not support self-dimerization. These models represent the different stoichiometries tested for the Atf7IP self-oligomerization; a) 2Atf7IP d) 3Atf7IP g) 4Atf7IP j) 5Atf7IP The right (b-c, e-f, h-i, k-l) of each panel contains the pLDDT-colored model predicted for each stoichiometry with its corresponding pLDDT plot.

Figure S5

```
Atf7IP          MDSLEEPQKKVFKARKTMRVSDRQQLEAVYKVKEELLKTDVKLLNGNHENGDLDPTSPLENMDYIKDKEEVNGIEEICFDPEGSKAEWKETPCILSVNVKNKQDDDLNCEPLSPHNITPE 120
Atf7IP2         ------------------------------------------------------------------------------------------------------------------------ 0
Atf7IP          PVSKLPAEPVSGDPAPGDLDAGDPASGVLASGDSTSGDPTSSEPSSSDAASGDATSGDAPSGDVSPGDATSGDATADDLSSGDPTSSDPIPGEPVPVEPISGDCAADDIASSEITSVDLA 240
Atf7IP2         ------------------------------------------------------------------------------MASPDRSKRKILKA--KKTMPL--SCRKQVEMLNKSRNVEA- 37
                                                                                              ::* * :. . : .    . *:  .*  :    .:  .*:
Atf7IP          SGAPASTDPASDDLASGDLSSSELASDDLATGELASDELTSESTFDRTFEPKSVPVCEPVPEIDNIEPSSNKDDDFLEKNGADEKLEQIQSKDSLDEKNK--ADNNIDANEETLE---TD 355
Atf7IP2         -----LKTAIGSNVPSG----------------------------NQSFSPSVIT---RTTEITKCSPSENGASSLDS------------NKNSISEKSKVFSQNCIKPVEEIVHSETKL 109
                      .   ..:: **                            :::*.*. :     . ** : .**.*  ..: .            .*:*:.**.*  ::* *.  ** :.   .
Atf7IP          DTTICSDRPPENEKKVEEDIITELALGEDAISSSMEIDQGEKNEDETSADLVETINENVIEDNKSE---------NILENT------------DSMETDEIIPILEKL-------APSED 447
Atf7IP2         EQVVCSYQKPSRTTESPSRVFTEEAK--DSLNT------SE-NDSEHQTN----VTRSLFEHEGACSLKSSCCPPSVLSGVVQMPESTVTSTVGDKKTDQMVFHLETNSNSESHDKRQSD 216
                : .:** : *.. .:  . ::** *   *::.:      .* *:.* .::    :...::*.: :          .:*...            .. :**:::  **.          ..*
Atf7IP          ELTCFSKTSLLPIDETNPDLEEKMESSFGSPSKQESSESLPKEAFLVLSDEEDISGEKDESEVISQNETCSPAEVESNEKDNKPEEEEQVIHEDDERPSEKNEFSRRKRSKSEDMDNVQS 567
Atf7IP2         NILCSEDSGFVPVEKTPN-LVNSVT------------------------------S-NNCADDILKTDECSRTSISNCESADSTWQ--SSLD------TNNNSHYQKKRMFSENEENVKR 296
                :: * ..:.::*:::*   * :.:                               . :: :: * :.: ** :.:.. *. :.  :  . :.      :::*.. ::**  **: :**:
Atf7IP          KRRRYMEEEYEAEFQVKITAKGDINQKLQKVIQWLLEEKLCALQCAVFDKTLAELKTRVEKIECNKRHKTVLTELQAKIARLTKRFEAAKEDLKKRHEHPPNPPVSPGKTVNDVNSNNNM 687
Atf7IP2         ---MKTSEQINENICVS----LERQTAFLEQVRHLIQQEIYSINYELFDKKLKELNQRIGKTECRNKHEGIADKLLAKIAKLQRRIKTVLLF--QRNCLKPNMLSSNGAS--KVANS--- 402
                      .*: : :: *.     : :  : : :: *::::: :::  :***.* **: *: * **.::*: :  :* ****:* :*:::.     :*:   **   * * :  .* ..
Atf7IP          SYRNAGTVRQMLESKRNVSESAPPSFQTPVNTVSSTNLVTPPAVVSSQPKLQTPVTSGSLTATSVLPAPNTATVVATTQVPSGNPQPTISLQPLPVILHVPVAVSSQPQLLQSHPGTLVT 807
Atf7IP2         --------EAMILD-KNL-----ES-------------------------VNSPIEKSSVNYE-----PS---------NPSEK--------------------GSKKINLSSDQNKSVS 449
                        . *: . :*:      *                         :::*: ..*:.       *.          ** :                    .*:   *.*. .. *:
Atf7IP          NQPSGNVEFISVQSPPTVSGLTKNPVSLPSLPNPTKPNNVPSVPSPSIQRNPTASAAPLGTTLAVQAVPTAHSIVQATRTSLPTVGPSGLYSPSTNRGPIQMKIPISAFSTSSAAEQNSN 927
Atf7IP2         ESNNDDVMLISVESPNLTTPITSNPTDTRKI-----------------------------------------------------------------------------------TSGNSS 486
                :. ..:* :***:**  .: :*.**..  .:                                                                                   :. **.
Atf7IP          TTPRIENQTNKTIDASVSKKAADSTSQCGKATGSDSSGVIDLTMDDEESGASQDPKKLNHTPVSTMSSSQPVSRPLQPIQPAPPLQPSGVPTSGPSQTTIHLLPTAPTTVNVTHRPVTQV 1047
Atf7IP2         NSPNAEVM----------------------AVQKKLDSIIDLTKE----------------------------------------------------------------------GLSNC 514
                .:*. *                        *. .. ..:**** :                                                                       :::
Atf7IP          TTRLPVPRAPANHQVVYTTLPAPPAQAPLRGTVMQAPAVRQVNPQNSVTVRVPQTTTYVVNNGLTLGSTGPQLTVHHRPPQVHTEPPRPVHPAPLPEAPQPQRLPPEAASTSLPQKPHLK 1167
Atf7IP2         NTESPV--------------------SPLESHSKAASNSKETTPLAQ--------------------------NAVQVPESFEHLPPLPEPPAPLPELV------DKTRDTLPPQKPELK 582
                .*. **                    :**..    *   ::..*  .                          .. : * ...  ** *  ******         :: .*  ****.**
Atf7IP          LARVQSQNGIVLSWSVLEVDRSCATVDSYHLYAYHEEPSATVPSQWKKIGEVKALPLPMACTLTQFVSGSKYYFAVRAKDIYGRFGPFCDPQSTDVISSTQSS  1270
Atf7IP2         VKRVFRPNGIALTWNITKINPKCAPVESYHLFLCHENSNN--KLIWKKIGEIKALPLPMACTLSQFLASNRYYFTVQSKDIFGRYGPFCDIKSIPGFSENLT-  682
                : **   ***.*:*.: ::: .** *:****:  **: .      ******:***********:**::..:***:*::***:**:***** :*   :*.. :
```

Figure S5 Clustal-O sequence alignment of Atf7IP and Atf7IP2.

The red box highlights the coiled-coil regions of Atf7IP and Atf7IP2. Abbreviations: * = identical residues, : = strongly similar residues, . = moderately similar residues

Figure S6

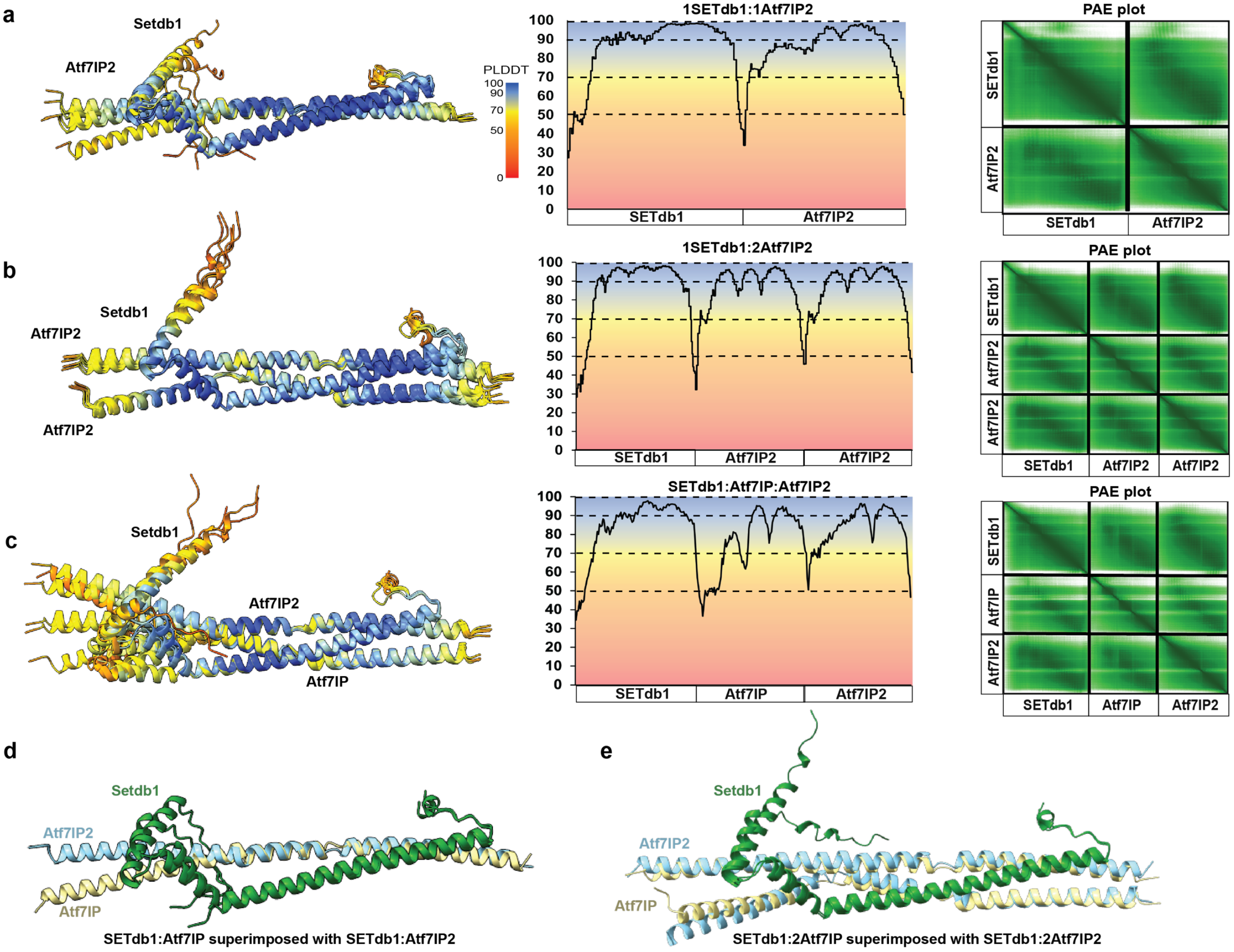

Figure S6 : Alphafold2 models of the minimal 1:1 and 1:2 Setdb1/Atf7IP2 complexes are similar to the m. a-b) Alignment of five top ranked models of the Setdb1(2-115)/Atf7IP2(297-388) 1:1 and 1:2 models colored by pLDDT scoring with their corresponding pLDDT plot and PAE plot of the highest ranked model c-d) Superimposed models of the 1:1 and 1:2 SETdb1/Atf7IP2 with the SETdb1/Atf7IP minimal complexes.

Figure S7

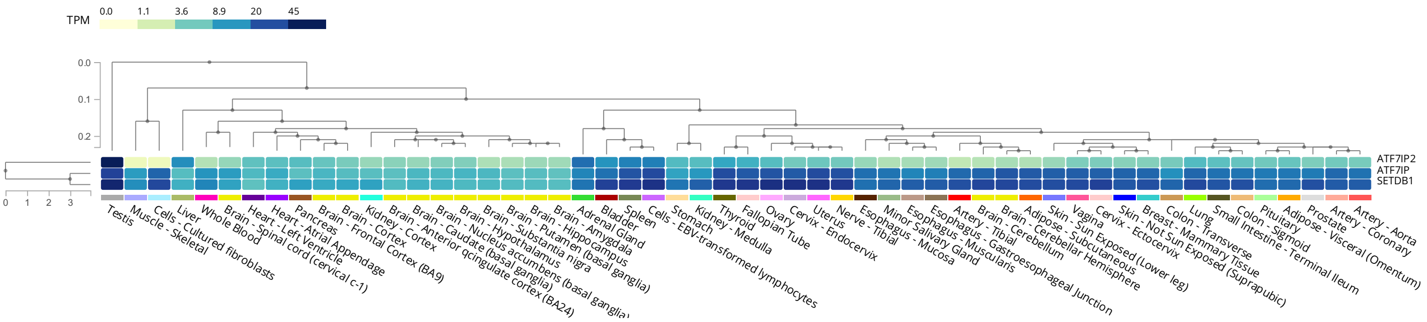

Figure S7: Differential expression of SETdb1, Atf7IP and Atf7IP2 in human tissues. This plot was generated using data from GTEx portal to compare the abundance of the Atf7IP paralogs in different tissues. TPM; transcripts per million.
